# Effect of aerobic exercise training on regional blood flow and vascular resistance in diabetic rats

**DOI:** 10.1186/s13098-015-0109-1

**Published:** 2015-12-21

**Authors:** Sarah Cristina Ferreira Freitas, Ângela d’Avila Harthmann, Bruno Rodrigues, Maria-Cláudia Irigoyen, Kátia De Angelis

**Affiliations:** Translational Physiology Laboratory, Universidade Nove de Julho, Rua Vergueiro 235/249, 2º subsolo, São Paulo, SP 01504 001 Brazil; Hypertension Unit, Heart Institute (InCor), School of Medicine, University of São Paulo, São Paulo, Brazil; Departament of Adapted Physical Education, Faculty of Physical Education (FEF), University of Campinas (UNICAMP), Campinas, SP Brazil

**Keywords:** Regional blood flow, Exercise training, Streptozotocin rats, Diabetes

## Abstract

**Background:**

Hyperglycemia has been associated with decreased blood flow in various organs, leading to tissue damage and dysfunctions. Exercise training (ET) is known to promote beneficial changes in the autonomic nervous system and may have effects on circulation. The aim of this study was to evaluate coronary and renal blood flows and vascular resistances after ET in diabetic rats.

**Methods:**

Thirty-two rats were divided into four groups (n = 8): sedentary control (SC), trained control (TC), sedentary diabetic (SD), trained diabetic (TD). Diabetes was induced by an injection of streptozotocin (STZ, 50 mg/kg). The ET was performed on a treadmill for 10 weeks. The blood flows were measured using colored microspheres.

**Results:**

The diabetic groups presented hyperglycemia (blood glucose >350 mg/dL) and ET did not change this parameter. The SD group showed reduced renal blood flow when compared to SC group, and ET was able to normalize this parameter in TD rats (SC: 4.3 ± 0.5; TC: 2.9 ± 0.3; SD: 1.9 ± 0.4; TD: 3.2 ± 0.4 mL/min/g). TD group presented increased coronary blood flow in relation to SD group (SC: 2.3 ± 0.23; TC: 2.8 ± 0.5; SD: 1.2 ± 0.4; TD: 3.0 ± 0.4 mL/min/g). The heart and kidneys vascular resistance were increased in SD group when compared to SC group, and ET was able to reverse these changes.

**Conclusions:**

Given the relevance of cardiomyopathy and nephropathy in mortality of diabetics, our results demonstrated that ET is effective in improving coronary and renal blood flows and vascular resistances in STZ-diabetic rats, reinforcing the positive role of this approach in preventing hyperglycemia-induced long-term organ damage.

## Background

Diabetes mellitus (DM) is now seen as a worldwide epidemic disease with high prevalence and incidence data [1]. The main cause of death in diabetic patients is due to cardiovascular disease [2]. This occurs because vascular disease is common in diabetes and it triggers major chronic complications throughout the body. These complications may arise from either microvascular (i.e., retinopathy, nephropathy, and neuropathy) or macrovascular disorders (i.e., cardiovascular disease, cerebrovascular accidents, and peripheral vascular disease) [3].

Impairment of blood flow in the brain, eye, heart, kidney, skeletal muscle, skin, and penile tissues have all been reported in type I and type II diabetes in experimental animals and humans [4]. The mechanisms leading to these alterations on blood flow are complex and have not yet been fully understood. They may be associated with both increased reactive oxygen and nitrogen species (RONS) production and endothelial dysfunction [4, 5].

Exercise training (ET), as in many other chronic diseases, has been recommended for the treatment of diabetic patients. Studies with experimental models of diabetes have shown that the hyperglycemia leads to an autonomic dysfunction characterized by reduced heart rate (HR) variability and impaired baroreflex sensitivity [6, 7]. Not infrequently, this dysfunction can be clinically detected in the diabetic population (both diabetes mellitus type I and II) [8] and it is associated with high rates of morbidity and mortality [9]. Our group has already shown that ET is effective in improving hemodynamic (arterial pressure and heart rate) and baroreflex and chemoreflex control of circulation in diabetic rats [6]. ET also attenuated autonomic and cardiac dysfunctions in this animal model [7]. Moreover, it is also known that the ET is able to improve endothelial function, as already demonstrated by the attenuating effects of ET on the decline of brachial artery flow-mediated dilatation in older trained women [10, 11]. In fact, many studies on exercise training in animals have been undertaken to provide a better understanding of ET physiology, ET beneficial effects and their underlying mechanisms, while several other research focused on establishing physical training protocols. Given that the autonomic nervous system is related to endothelial cells [12], inflammation [13], and renin–angiotensin–aldosterone system [14], affecting circulation and circulation control, we hypothesize that ET improves regional blood flows, causing positive changes on regional vascular resistances in streptozotocin (STZ) diabetic rats. Thus, the aim of the present study was to evaluate regional blood flow (RBF) and regional vascular resistance (RVR) after ET in streptozotocin-induced diabetic rats.

## Methods

### Animals

Male Wistar rats were used for the experiments, weighing approximately 250–300 g from the animal house of the University of São Paulo. The animals were housed in plastic boxes containing four animals per cage, with temperature-controlled room between 22 and 24 °C and a 12 h light–dark cycle. Chow and water were given “ad libitum”. After the surgical procedures, the animals were kept in individual cages. All surgical procedures and protocols used were in accordance with the Guidelines for Ethical Care of Experimental Animals approved by the International Animal Care and Use Committee. This study protocol was approved by the Ethic Committee of Universidade de Sao Paulo (Protocol 2134/02/141). The animals were randomly assigned to one of four groups: sedentary control (SC, n = 8), trained control (TC, n = 8), sedentary diabetic (SD, n = 8) and trained diabetic (TD, n = 8).

### Diabetes induction

At the start of the protocol, animals of SD and TD groups were made diabetic by a single injection of STZ (50 mg/kg, iv, Sigma Chemical Co., St. Louis, MO, USA) dissolved in citrate buffer, pH 4.5. Rats were fasted overnight before STZ injection.

### Exercise training

Low intensity exercise training (40–60 % maximal running speed) was performed 1 week after diabetes induction on a treadmill twice a day (1 h each time), 5 days a week for 10 weeks, as described in detail elsewhere [7]. All animals were adapted to the procedure (10 min/day; 0.3 km/h) for 1 week before beginning the exercise training protocol. This adaptation period began 24 h after diabetes induction. Sedentary and trained groups underwent a maximal treadmill test as previously described in detail [15]. The tests were performed: (1) at the beginning of the experiment and (2) on the fifth and (3) tenth week of the training protocol to determine exercise capacity and exercise training intensity.

### Metabolic evaluations

Body weight was monitored weekly during the period of physical activity. Blood samples were collected from the rats at rest, in the fasting state, 1 week and 10 weeks after diabetes induction. Plasma glucose was measured by a colorimetric enzymatic test (Enz Color, Bio Diagnostica, Piraquara, Paraná, Brazil).

### Hemodynamic measurements

Twenty-four hours after the blood samples were collected (11th week) a catheter filled with 0.06 mL of saline was implanted into the femoral artery of the anesthetized rats (80 mg/kg ketamine and 12 mg/kg xylazine, ip). A second catheter of PE-50 was inserted into the left ventricle through the right carotid artery for colored microsphere infusion. The catheter position was determined by observing the characteristics of left ventricular pressure waveform at surgery, and confirmed at necropsy.

The femoral arterial cannula was connected to a strain-gauge transducer (P23Db, Gould Statham, Oxnard, CA, USA) and blood pressure signals were recorded over a 10-min period by a microcomputer equipped with an analog-to-digital converter board (CODAS, 1-kHz sampling frequency, Dataq Instruments, Inc, Akron, OH, USA). The recorded data were analyzed on a beat-to-beat basis to quantify changes in arterial pressure (AP) [16].

### Microsphere infusion

Yellow (300,000/180 μL) 15-mm dye extraction Dye-Trak microspheres were obtained from Triton Technology (San Diego, CA, USA) and infused after 10 weeks of exercise training. Microsphere infusion and processing were undertaken in accordance with previous descriptions [17, 18]. Vials of commercial stocks of microsphere infusates were sonicated for 5 min and inverted several times immediately before dilution to the desired concentration with 0.9 % saline solution containing 0.01 % Tween 80. To determinate average dye content of the microspheres, 200 μL of the diluted commercial suspensions were taken after 5 min of sonication. These aliquots were placed in 15-mL tubes and were centrifuged, dried, and extracted with dimethylformamide. The mean absorbance was determined by a spectrophotometer and divided by the average microsphere concentration to obtain an average dye concentration [absorbance units (spheres mL^−1^)^−1^] [19]. Sphere dilutions were sonicated for 5 min before infusion. A coil of PE-50 tubing (75 cm) was then filled with 180 μL of the infusate of colored microspheres and interposed between the left ventricular catheter and a 1-mL syringe containing 0.5 mL of pre warmed (40 °C) saline solution. This syringe was mounted on a variable-speed infusion pump (Infusion Pump 22, Harvard Apparatus, South Natick, USA). With a withdrawal pump (Infusion and Withdrawal Pump, Harvard Apparatus, South Natick, USA), reference blood samples were drawn from the abdominal aorta catheter at the rate of 0.5 mL/min into a pre heparinized and weighed with an 1-mL disposable syringe. The withdrawal of a reference blood sample started 10 s before the beginning of microsphere infusion and was continued for 75 s. The microspheres and the saline solution were infused for 50 s at a rate of 0.36 mL/min. After microsphere infusions, the animals were killed by sodium pentobarbital overdose.

### Regional blood flow and vascular resistance evaluation

Heart, lungs, kidneys and liver were removed to determine regional blood flow. Reference blood and tissues were processed as described in detail elsewhere [17, 18]. The reference blood samples and tissues were processed as described by De Angelis et al. [17]. To extract the dyes from the isolated dried microspheres, 500 µL of dimethylformamide was added to each tube, which was briefly but vigorously vortexed. The samples were centrifuged (2000*g*, 10 min) and the absorbance of the supernatant was determined with a DU 640 spectrophotometer (Beckmann Instruments, Inc, Fullerton, CA, USA; <1.8 nm slit width) using a 700 µL quartz cuvette. The absorption spectrum peaks for the yellow microspheres were obtained at 448 nm, respectively. The absorbance values were corrected using a matrix inversion technique (Dye-Trak matrix inversion macro for Excel, Triton Technology, San Diego, CA, USA). The minimum acceptable absorbance was 0.010 AU. For each infusion, the tissue flow rates were calculated according to the following formula [18]: Qt = At(Qb/Ab); where Qt and Qb stand for the flow in the sample tissue and in the reference blood, respectively, and At and Ab represent stand for the peak absorbance (AU) of the tissue sample and of the reference blood, respectively. Qb, in mL/min, was calculated by:

$${\text{Qb}} = \frac{{{\text{Reference blood sample weight/1}}.0 5 \;{\text{g}}/{\text{mL}}}}{{{\text{Reference blood sample volume}}/0. 5 \;{\text{mL}}/{ \hbox{min} }}}$$

where 1.05 g/mL is the specific gravity of blood and 0.5 mL/min is the withdrawal rate. Blood flow rates were divided by tissue weights to yield mL/min/g. Cardiac output (CO) was calculated by the following formula [19]:

$$\frac{{{\text{Total number of injected microspheres }} \times {\text{ reference rate }}\left( {0. 5 \,{\text{mL}}/{ \hbox{min} }} \right)}}{\text{Number of microspheres in the reference blood sample}}$$

Cardiac output divided by the body weight of each animal was defined as the cardiac index. Peripheral vascular resistance (PVR) was calculated as mean AP divided by the cardiac index (mmHg/mL/min/kg). Regional vascular resistance (RVR) was calculated as mean AP divided by regional tissue flow (mL/min/g).

### Statistical analyses

Data are reported as mean ± SEM. The Levene Test was used to evaluate data homogeneity and two ways ANOVA was used to compare groups, followed by the Student–Newman–Keuls test. Significance level was established at P < 0.05.

## Results

### Body weight and glycemia

The TC group had a lower body weight when compared to SC group. Body weight of both diabetic groups (SD and TD groups) was reduced when compared to control animals (SC and TC groups); however, exercise training increased body weight in TD group when compared to SD group (Table 1).

**Table 1:** Metabolic evaluations and exercise capacity of the studied groups

	SC	TC	SD	TD
Body weight (g)	379 ± 11	338 ± 10*	254 ± 13*	295 ± 11*^,#,†^
Glycemia (mg/dL)	152 ± 80	160 ± 20	351 ± 29*	345 ± 11*
Maximal running test (km/h)	1.0 ± 0.07	2.2 ± 0.02*	0.9 ± 0.19	2.0 ± 0.02*^,#^

Data are reported as mean ± SEM. Sedentary control (SC, n = 8), trained control (TC, n = 8), sedentary diabetic (SD, n = 8), trained diabetic (TD, n = 8)

* P < 0.05 vs. SC; ^#^ P < 0.05 vs. SD; ^†^ P < 0.05 vs. TC

Unlike the control groups, which received only citrate buffer (SC and TC groups) (Table 1), the diabetic groups presented hyperglycemia (SD and TD groups).

### Exercise capacity

Exercise capacity was lower in sedentary animals (SC and SD groups) in relation to the trained groups (TC and TD groups) after 10 weeks of exercise training (Table 1).

### Hemodynamic parameters

A reduction in both mean AP and CO was observed in the SD group when compared to the SC group. These values were normalized in the TD group, reaching values similar to the SC group. Cardiac index was similar in the studied groups, except for the TC group, which showed higher values when compared with SD group. PVR and TPVR values were similar in the studied groups (Table 2).

**Table 2:** Hemodynamics evaluations of the studied groups

	SC	TC	SD	TD
Mean arterial pressure (mmHg)	109 ± 2	109 ± 3	93 ± 3*	105 ± 2^#^
Cardiac output (mL/min)	92 ± 7	107 ± 8	55 ± 5*	76 ± 7
Cardiac index (mL/min/kg)	267 ± 19	319 ± 33	192 ± 15^†^	275 ± 35
PVR (mmHg/mL/min)	1.23 ± 0.22	1.14 ± 0.16	1.65 ± 0.22	1.6 ± 0.2
TPVR (mmHg/mL/min/kg)	0.36 ± 0.05	0.42 ± 0.05	0.48 ± 0.05	0.38 ± 0.04

Data are reported as mean ± SEM. Sedentary control (SC, n = 8), trained control (TC, n = 8), sedentary diabetic (SD, n = 8), trained diabetic (TD, n = 8)

*PVR* peripheral vascular resistance, *TPVR* total peripheral vascular resistance

* P < 0.05 vs. SC; ^#^P < 0.05 vs. SD; ^†^ P < 0,05 vs. TC

### Regional blood flows

**Fig. 1:**
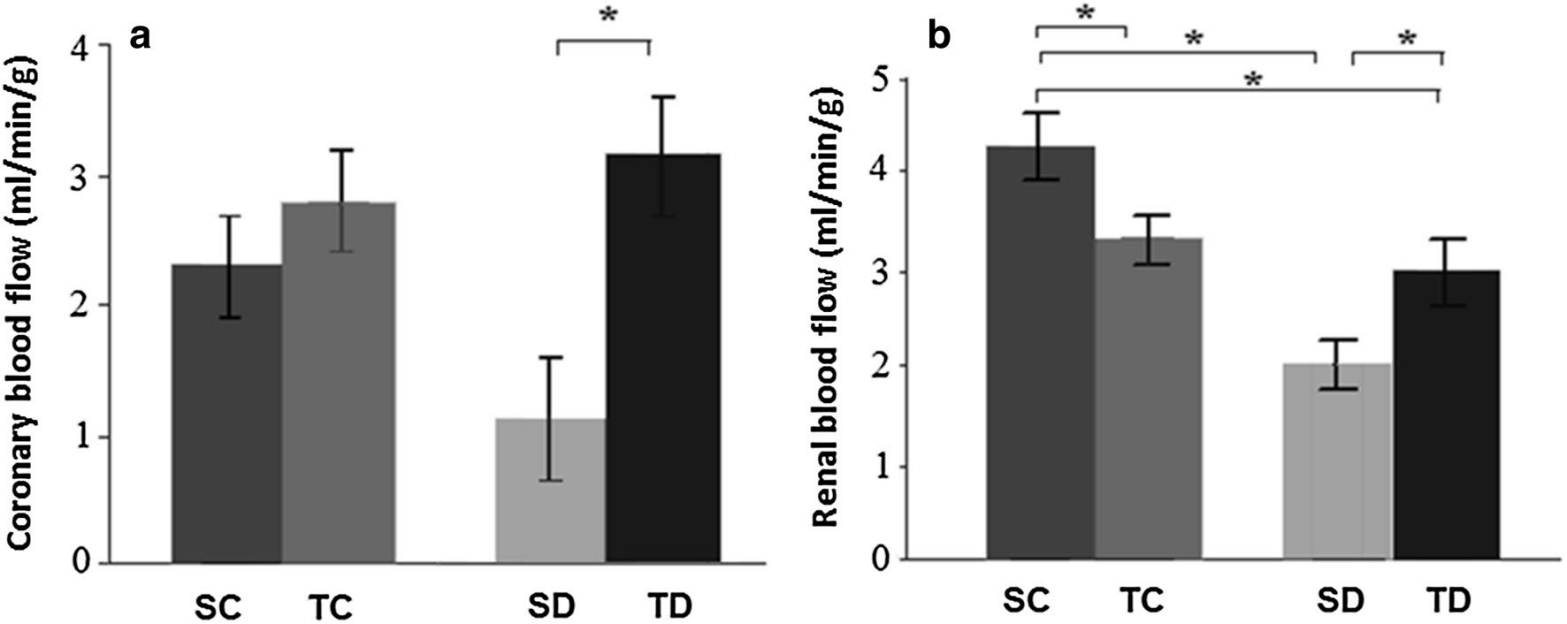
**a** Coronary blood flow and **b** renal blood flow. *P < 0.05. Sedentary control (SC, n = 8), trained control (TC, n = 8), sedentary diabetic (SD, n = 8), trained diabetic (TD, n = 8). Data are reported as mean ± SEM

Coronary blood flow was similar in both SC (2.3 ± 0.2 mL/min/g) and TC groups (2.8 ± 0.5 mL/min/g). There was an increase in this parameter in TD (3.0 ± 0.4 mL/min/g) when compared to SD group (1.2 ± 0.4 mL/min/g) (Fig. 1a).

Diabetes (SD group) induced a decrease in renal blood flow when compared to SC group. Exercise training (TD group) improved this flow (vs. SD group), but TD group showed reduced renal blood flow in relation to SC group (SC: 4.3 ± 0.5; TC: 2.9 ± 0.3; SD: 1.9 ± 0.4; TD: 3.2 ± 0.4 mL/min/g) (Fig. 1b).

Pulmonary blood flows were similar in all groups (SC 1.63 ± 0.30; TC: 1.85 ± 0.44; SD: 1.61 ± 0.57; TD: 2.02 ± 0.43 mL/min/g).

The exercise training was effective in increasing hepatic blood flow only in TC group (0.86 ± 0.17 mL/min/g) in relation to other groups (SC: 0.20 ± 0.05; SD: 0.17 ± 0.04 and TD: 0.22 ± 0.04 mL/min/g).

### Regional vascular resistances

**Fig. 2:**
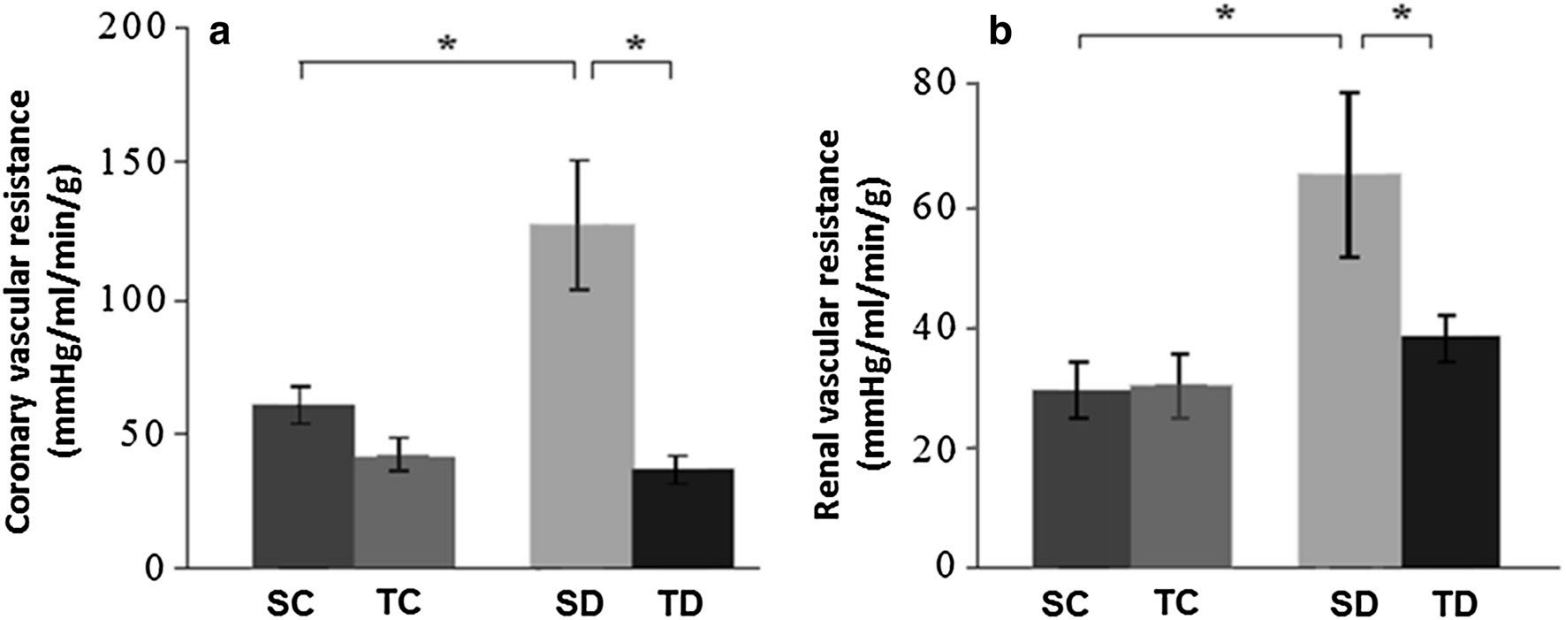
**a** Coronary vascular resistance and **b** mean renal vascular resistance. *P < 0.05. Sedentary control (SC, n = 8), trained control (TC, n = 8), sedentary diabetic (SD, n = 8), trained diabetic (TD, n = 8). Data are reported as mean ± SEM

Coronary and renal vascular resistances presented an increase in SD group (heart: 127 ± 24 and kidneys: SD: 65 ± 14 mmHg/mL/min/g) when compared to SC group (heart: 59 ± 7 and kidneys: 30 ± 5 mmHg/mL/min/g). Exercise training in diabetic rats (TD group) was able to normalize these regional resistances (heart: 36 ± 5 and kidneys: 38 ± 4 mmHg/mL/min/g). There were no significant differences between SC and TC groups (TC heart: 41 ± 6 and kidneys 30 ± 6 mmHg/mL/min/g) (Fig. 2a, b).

## Discussion

Long-term complications of diabetes occur due to the abnormal metabolic state, hemodynamic modifications, and other still poorly characterized factors [20]. In this study we showed a reduction of coronary and renal blood flows in an experimental model of STZ-induced diabetes and without relevant impact on pulmonary and hepatic blood flows, thus demonstrating the impact of hyperglycemia on cardio-renal systems. Importantly, ET may reverse the impairment of circulation in these organs, even if the hyperglycemic status remains unchanged.

Polyphagia is observed clinically in diabetic humans [3]. In the present study we did not measured food intake; however it is well-established that the diabetic animal model eats more than control, although their body weight is reduced, because they gain less weight with time than control animals [6, 7, 21]. The reduced body weight gain in STZ-diabetic rats, confirmed in the present study, is probably related to lipid and protein consumption as energy substrates, since the use of glucose as an energetic substrate is very difficult in diabetic states. Considering this condition, we applied a protocol of training in which STZ-diabetic rats underwent two sessions/day of aerobic exercise [6, 7]. This frequency of training aimed to promote two opportunities of glucose transporter (GLUT4) translocation to the muscle membrane during the day, increasing the possibility to improve metabolic/glycemic control. Exercise training was able to increase the body weight of STZ-diabetic rats (TD vs. SD), indicating an improvement of the metabolic state, but body weight was reduced when compared to both control groups (SC and TC). However, no alteration on glycemia was found when comparing the initial and final values of the same group, showing that aerobic ET was not able to lower blood glucose in this model of diabetes.

It is interesting to note that increased hepatic blood flow after training was observed in normal animals, which is probably linked to the role of the liver as a glucose provider organ for skeletal muscle metabolism during exercise. Thus, the liver could be seen as a sensor whose function is to monitor metabolic regulation during exercise [22]. However, those changes in hepatic blood flow were not observed in trained diabetic animals, suggesting that alternative forms of energy supply (such as free fat acids) to the muscle during exercise may account for this different adaptation to training.

ET was able to improve the exercise capacity, as shown by the higher maximal velocity reached in the exercise test by both trained groups (TC and TD). The result of maximal velocity test in treadmill is correlated with oxygen consumption [15], indicating that the protocol intensity was effective in promoting fitness.

Furthermore, ET normalized the reduction of AP observed in STZ-induced diabetes rats, recovering control values, as described elsewhere [6]. It has been argued that the change in AP in diabetic rats may be due to peripheral vascular resistance alteration [23], but in our study we found no differences in total peripheral vascular resistance among groups. Our evaluation of regional flows using microspheres showed a reduction in some territories and no changes in others, thus suggesting that reducing flows in some tissues may be reflected in TPVR. Both coronary and renal flow resistance were increased; however, although TPVR is believed to increase in sedentary diabetic animals, the values we found had no statistical significance. It is therefore possible that other areas in which we did not measure blood flow (such as muscles) were changed, and this may explain why we did not find major differences in peripheral resistance.

Jackson and Carrier [24] have also suggested that decreased AP was due to reduced cardiac output in sedentary diabetic rats, because these rats develop a hypovolemia caused by hyperglycemic osmotic diuresis. In the present study, we observed reduced CO in SD group when compared to SC group, and ET was able to increase cardiac output, as previous studies have demonstrated [25]. In fact, we have previously observed reduced systolic and diastolic functions evaluated by catheterization and by echocardiogram in vivo [26], as well as in isolated hearts in vitro in STZ-diabetic rats [7]. Additionally, Silva and colleagues [27] have demonstrated that ET was able to improve ejection fraction and isovolumetric relaxation time in STZ-diabetic rats, suggesting improvement in CO in trained diabetic rats, just as observed in the present study.

In our study, the pulmonary and hepatic blood flows of the SD group remained within the normal range when compared to sedentary controls. It is known that eNOS levels in lungs of STZ-induced diabetic rats is increased [4, 28], suggesting that they may prevent endothelial dysfunction in this organ. However, renal flow was reduced (SD vs. SC) and coronary blood flow, although lower in diabetics, was similar in these two groups. Interestingly, regional vascular resistance was increased in coronary and renal circulations in the diabetic sedentary rats (vs. SC), and this suggests that important regional vascular resistances were increased and may be associated with the end organ damage observed in diabetics.

Other researchers have also described the impaired renal blood flow observed in our study. Hill and Larkins [29], using radioactive microspheres, have found decreased renal blood flow 2–4 weeks after diabetes induction. However, other studies have pointed to increased renal blood flow [30]. Some have suggested that the increased production of nitric oxide (NO) may be associated with this increase in renal blood flow [31]. However, several studies have shown that although diabetes induces greater production of NO, it also increases the oxidation of NO by superoxide anion. The balance of these two systems determines the effect of NO on renal vascular tone [4]. Therefore, although diabetic animals show increased urinary excretion of nitrites and nitrates (NO metabolites), there seems to be greater need of NO production to maintain the minimum infusion in renal microvasculature. This mechanism, if present, may be responsible for the maintenance of the minimum tissue perfusion in diabetic state. This hypothesis concurs with the possibility of a maintained renal vasoconstriction status in diabetes, as observed in other peripheral areas [32]. Another way to interpret the reduction of renal blood flow in experimental diabetes would simply assign it to the lowest CO or CI in these animals [33].

Another interesting point is that changes in regional blood flows do not seem to be simply related to one another or variations of CO or CI. They could also represent an adaptation of certain tissues metabolic disorders. In this aspect, hyperglycemia could produce such a result, since glucose may have a direct vasoconstrictor potential [34]. In fact, glucose increase may lead to increased production of RONS. This would reduce the availability of NO and induce a state of increased vasoconstrictor tone, thus contributing to left ventricular instability [35]. In our study, although there was no significant reduction of coronary flow in SD group when compared to their respective controls using analysis of variance, a direct comparison of these values through the Student t test (assessing therefore only the effect of diabetes) showed a significant reduction in coronary blood flow induced by experimental diabetes (P < 0.05). Furthermore, the increased vascular resistance to this territory observed in STZ diabetic rats reinforces the possibility of vasoconstriction in this territory. In fact, many studies in the literature have shown a high incidence of macro and microvascular disease in diabetic patients [3] and among the pathophysiological mechanisms, the endothelium seems to have an important role. Moreover, a study has shown that the dysfunction of the endothelium-dependent vasodilator response, determined by coronary flow, is proportional to cardiac sympathetic dysfunction [36].

It is important to highlight that the most relevant result of this study was the effects of exercise training on coronary and renal blood flows and vascular resistance, which had not been documented in the literature. In this sense, the renal blood flows reverse to normality values, and as such this maybe directly related to the improvement in CO observed in trained diabetic animals. ET has already shown beneficial effects in renal function in STZ-diabetic rats [27], and this may be associated with the improved blood flow and vascular resistance in this tissue after training observed in the present study. Interestingly, despite normal renal vascular resistance, both trained groups (TC and TD) presented decreased renal blood flow when compared to the control group. McAllister concluded in an elegant review that studies of mechanisms involved in the changes in renal blood flow in healthy subjects suggest that reduced sympathetic nervous system outflow, plasma angiotensin II and vasopressin concentrations are involved in lesser splanchnic and renal vasoconstriction observed by trained subjects. In addition, the author reported that such changes may be related to the fact that splanchnic and renal blood flows are less reduced from resting levels during acute exercise after a period of endurance exercise training [37].

Furthermore, coronary flow was higher in trained diabetic rats than in sedentary diabetics. Changes in vascular reactivity induced by exercise training have been described in the literature. In a review, Lash [38] discussed these adaptations in coronary vessels suggesting that they depend on the size of the vessel. These observed chronic changes could also be due to changes related to the endothelium. Studies have indicated that endothelium-dependent coronary arterial vasodilator is normal in trained dogs [39]. However, Lash [38] suggests that vasodilation is increased early in the process of adaptation to exercise, but revert to normal once this adaptation is established, or rather, when structural changes lead to a reduction in coronary “shear stress” during exercise. Moreover, the improvement in coronary flow and resistance after training may be due to autonomic and metabolic changes brought about by ET. This in turn leads to a reduced oxidative stress in the heart, as demonstrated in diabetic ovariectomized aerobically trained rats [40–42], and may attenuate endothelial dysfunction.

## Conclusions

Given the relevance of diabetic cardiomyopathy and nephropathy in morbi-mortality of diabetic population, the results of the present study demonstrated that ET is effective in improving coronary and renal blood flows and vascular resistances in STZ-diabetic rats, reinforcing the positive role of this approach in preventing hyperglycemia-induced long-term organ damage.

## Abbreviations

ANS: autonomic nervous system
AP: arterial pressure
CO: cardiac output
DM: diabetes mellitus
ET: exercise training
HR: heart rate
PVR: peripheral vascular resistance
RBF: regional blood flow
RVR: regional vascular resistance
RONS: reactive oxygen and nitrogen species
SC: sedentary control
SD: sedentary diabetic
STZ: streptozotocin
TC: trained control
TD: trained diabetic
